# Use of Myometrium as an Internal Reference for Endometrial and Cervical Cancer on Multiphase Contrast-Enhanced MRI

**DOI:** 10.1371/journal.pone.0157820

**Published:** 2016-06-21

**Authors:** Chia-Ni Lin, Yu-San Liao, Wen-Chang Chen, Yue-Sheng Wang, Li-Wen Lee

**Affiliations:** 1 Department of Diagnostic Radiology, Chang Gung Memorial Hospital, Chiayi, Taiwan; 2 Department of Chemistry and Biochemistry, National Chung Cheng University, Chiayi, Taiwan; 3 Department of Medical Imaging and Radiological Science, Central Taiwan University of Science and Technology, Taichung, Taiwan; 4 Department of Nursing, Chang Gung University of Science and Technology, Chiayi Campus, Chiayi, Taiwan; University of Chicago, UNITED STATES

## Abstract

**Background:**

Myometrial smooth muscle is normally within the field of view for the gynecological imaging. This study aimed to investigate the use of normal myometrium as an internal reference for endometrial and cervical cancer during multiphase contrast-enhanced magnetic resonance imaging (MCE-MRI) and to explore whether this information regarding tumor enhancement relative to the myometrium could be used to discriminate between endometrial and cervical cancer.

**Methods:**

MRI images, before and after contrast enhancement, were analyzed in newly diagnosed cervical (n = 18) and endometrial cancer (n = 19) patients. Signal intensities (SIs) from tumor tissue and non-neoplastic myometrium were measured using imaging software.

**Results:**

The relative signal for cervical cancer was approximately 30% higher than that of endometrial cancer after contrast administration. The area under receiver operating characteristic curve for SI, relative signal enhancement, and tumor to myometrium contrast ratio (as used to discriminate between cervical cancer and endometrial cancer) was 0.7807, 0.7456 and 0.7895, respectively. There was no difference in SI of the normal myometrium between endometrial and cervical cancer patients prior to and after contrast administration. Using non-tumorous myometrium as an internal reference, the tumor to myometrium contrast ratio was significantly higher in tumor tissue from cervical cancer compared with that from endometrial cancer at 25 s post contrast enhancement (p = 0.0016), with an optimal sensitivity of 72.22% and specificity of 84.21%.

**Conclusion:**

With SI normalized to baseline or normal myometrium, tumor tissue from cervical cancer patients showed significant hyperintensity compared with that of tumor tissue from endometrial cancer patients after contrast enhancement, yielding acceptable performance. The use of the myometrium as an internal reference may provide an alternative method to analyze MCE-MRI data.

## Introduction

Cervical and endometrial cancers are common malignancies affecting the female genital tract. According to the World Cancer Report 2014, cervical cancer is the fourth most frequent cancer in women and endometrial cancer is the sixth most frequent cancer in women. Cervical cancer is the most common gynecologic malignancy arising from the junction between the squamous and columnar epithelium of the cervix, also known as the squamocolumnar junction (SCJ) [1, 2]. The location of the SCJ in the cervix is influenced by age and hormonal status [3]. In elderly patients, the SCJ is located within the cervical canal and cervical cancer in these patients may grow inward along the cervical canal. Therefore, the endocervical canal is a site from which both cervical and endometrial adenocarcinoma can arise. When a bulky tumor is present in both endometrial and cervical biopsies or when the precise site used to obtain the biopsy is unclear, it can be difficult to distinguish whether the mass is of cervical or endometrial origin for high grade carcinomas [4].

Immunohistochemical stains can help differentiate between tumors of cervical vs. endometrial origin [5]. However, there may be insufficient tissue to provide a definitive diagnosis in small samples. In such situations, magnetic resonance imaging (MRI) may assist in determining the primary site of cancer [4, 6]. Currently, MRI is the imaging modality of choice for staging and post-therapy surveillance in endometrial and cervical cancer [7–9]. Multiphase contrast-enhanced MRI (MCE-MRI) has become a popular MR sequence for staging endometrial cancer since its introduction by Yamashita et al. [10] in 1993 and is one of the suggested MRI protocols for both endometrial and cervical cancer by the European Society of Urogenital Radiology [7, 8].

For endometrial cancer, the tumor demonstrates weaker enhancement compared with the normal myometrium, with an optimal tumor to myometrium contrast which ranges between 90 and 150 s after contrast enhancement [8, 11]. For cervical cancer, MCE-MRI obtained 30–60 s after contrast injection is useful for identifying small tumors which are not seen on T2-weighted (T2W) images, as they can show increased early enhancement relative to the cervical stroma [12].

In clinical practice, the signal intensity (SI) of cervical cancer is usually compared with that of the cervical stroma whereas the SI of endometrial cancer is usually compared with that of the myometrium. Since cervical and endometrial cancer can both occur within the uterus and the cervix, there is a need to use the same internal reference in studies involving comparison of signal enhancement between both cancers. In theory, the uterine myometrium is larger than the cervix and may act as a better reference. This study aimed to investigate the use of the myometrium as an internal reference for both endometrial and cervical cancer on MCE-MRI and also to explore whether this information regarding tumor enhancement relative to the myometrium could be used to discriminate between endometrial and cervical cancer.

## Materials and Methods

### Subjects

**Fig 1:**
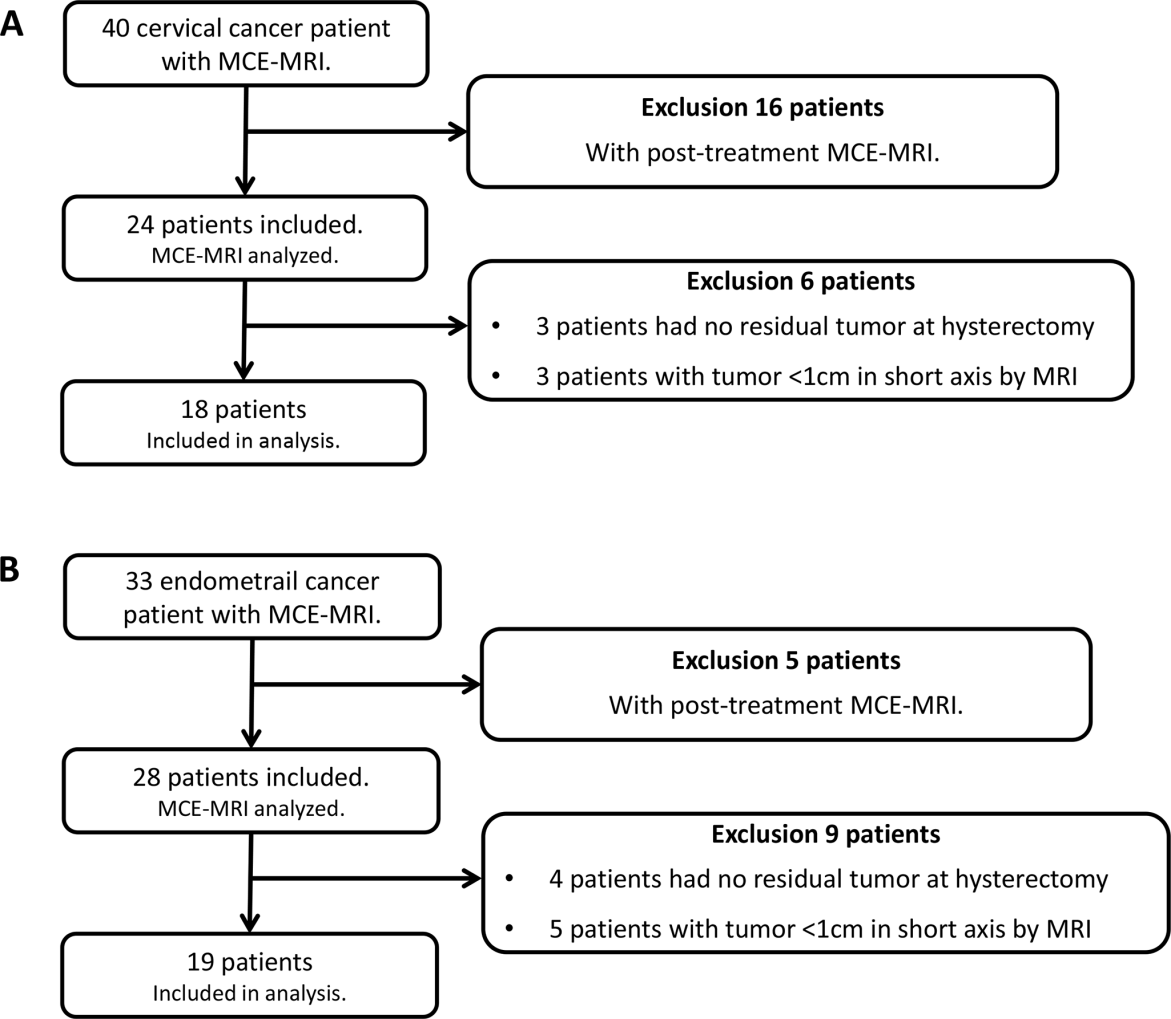
Patient selection for (A) cervical cancer and (B) endometrial cancer using multiphase contrast-enhanced magnetic resonance imaging (MCE-MRI).

This retrospective study was approved by the local institutional review board of the Chang Gung Memorial Hospital (103-2240B) and a waiver of informed consent was obtained. From June 2012 to February 2015, all adult women with histopathologically-proven primary endometrial or cervical cancer, and who received pelvic MRI including structural and MCE-MRI at our institute, were included in this study. Exclusion criteria included subjects who had received prior cancer treatment and who had a non-measurable tumor by MRI (< 5 mm on short axis on sagittal images). The flow diagram of patient selection is shown in **Fig 1**.

### Imaging protocol

Imaging was performed using a 3T Siemens Verio scanner equipped with software Syngo MR B17 (Siemens Medical System, Erlangen, Germany) using a six-channel body coil. Each patient was required to fast for at least 4 h prior to the scan. Each patient was also asked to empty her bladder before undergoing the MRI scan and to perform shallow breathing throughout the entire scan. To minimize motion artifact from bowel peristalsis, Buscopan 20mg (Hyoscine-M-Butylbromide, Nang Kuang Pharmaceutical, Tainan, Taiwan) was routinely given intravenously before MRI, unless contraindicated. The imaging protocol included both morphological and functional imaging (**S1 Table**). The morphological MRI protocol included axial T1-weighted (T1W) and T2W images, with a large field of view, to evaluate the entire pelvis. High-resolution T2W and diffusion-weighted images, in axial and sagittal planes, were used to evaluate the primary tumor.

The MCE-MRI protocol consisted of four sagittal acquisitions, at four different time points, using the fast low angle shotMRI pulse sequence. The parameters were as follows: repetition time = 4.32 ms, echo time = 1.59 ms, field of view = 19.5× 24.0 cm^2^, matrix size = 320 x 182, and number of acquisitions = 1. A total of 40 slices were obtained using a slice thickness of 3 mm and an acquisition time of 26 s. The 1^st^ scan was acquired prior to the injection of contrast media. Sampling times for the 2^nd^, 3^rd^ and 4^th^ scans were 25 s, 71 s and 141 s, respectively, after the start of the contrast injection. Gadolinium diethylenetriamine pentaacetic acid (Magnevist, Bayer Schering Pharma AG, Berlin, Germany) was administered intravenously (0.2 mmol/kg of body weight) with an MR-compatible power injector (Optistar^TM^ Elite Injector, Covidien, Cincinnati, OH, USA). T1W axial images with a large field of view were also acquired to evaluate the entire abdomen and pelvis.

### Imaging analysis

**Fig 2:**
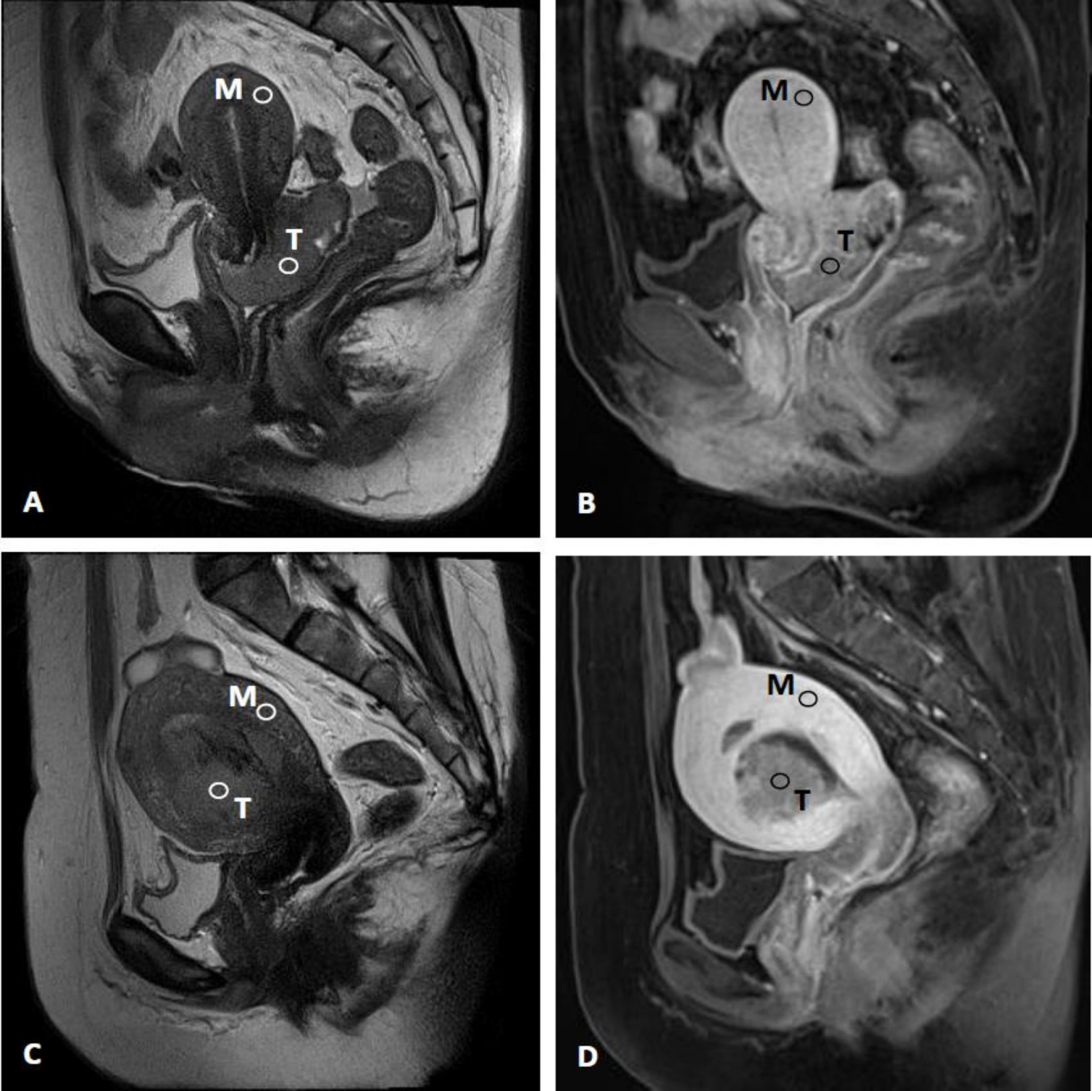
Illustration of region of interest placement in patients with (A,B) cervical cancer and (C,D) endometrial cancer on sagittal T2-weighted (left column) and contrast-enhanced T1-weighted (right column) MRI images. Abbreviations: T, tumor; M, myometrium.

Each region of interest (ROI) was drawn using Image J (Image J 1.3.1, NIH, USA) by two experienced radiologists with 10 years and 14 years of experience, respectively, in pelvic imaging. The two radiologists, blinded to the pathology results, performed the ROI drawing separately. A single ROI for tumor tissue in each patient was manually drawn from the enhanced portion of the tumor on the 4^th^ dynamic scan, avoiding the heterogeneous and necrotic regions, with reference to the T2W and contrast-enhanced T1W images. That ROI was then copied and pasted on the same slice location for all time points (**Fig 2**). In the event of significant motion, it may have been necessary to adjust the ROI position but the ROI was held to the same size and shape throughout the dynamic scans. ROI selection for the normal myometrium was based on the same slice using a similar method as that used for tumor tissue. If the normal myometrium was not measurable on the same slice used for the selected ROI of tumor tissue, the normal myometrium closest to the tumor ROI on an adjacent slice was measured. The ROI for normal myometrium was selected from the uniform central region of the outer myometrium and away from the edge to avoid partial volume effects (**Fig 2**). The tumor to myometrium contrast ratio (SI_T/M_) was calculated as SI of tumor divided by SI of the myometrium.

The relative signal enhancement (SI_relative_) was calculated as:

$${SI}_{relative}(\%)=\frac{{SI}_{t}-{SI}_{0}}{{SI}_{0}}\times 100\%$$

where SI_t_ is the SI following contrast administration and SI_0_ is the pre-contrast SI.

### Statistical analysis

PRISM 6 (GraphPad Software, Inc., San Diego, CA, USA) was used for data analysis and alsoto generate graphs. The SI and relative SI within each ROI were plotted over time. All data were expressed as mean ± standard error of the mean (SEM). The *t*-test was used to compare the means of two groups. A p-value < 0.05 was considered statistically significant. The Bland-Altman method for comparing paired measurements was used to determine interobserver agreement. The area under a receiver-operating-characteristic curve (ROC) was used to quantify the overall ability of the MR signal to discriminate between cervical and endometrial cancer. Optimal cut-off was defined as the point on the ROC curve which was farthest from the line of equality (Youden index).

## Results

Of the 73 patients identified, 52 patients with newly diagnosed cervical or endometrial cancer met the inclusion criteria. Fifteen patients had non-measurable lesions on MRI, leaving 18 patients with cervical cancer and 19 patients with endometrial cancer for the final analysis **(Fig 1)**. The patient demographics and tumor profiles are shown in **Table 1**.

**Table 1:** Patient characteristics and histopathological classification.

	Endometrial cancer	Cervical cancer
Number of subjects	19	18
Age (years), mean (range)	57.9 (36–85)	54.7 (35–86)
Tumor size, mean ± SEM (range)	5.4 ± 0.59 cm (2.3–14.0)	4.4 ± 0.42 cm (2.0–9.3)
Endometrioid carcinoma	14	0
Clear cell carcinoma	2	0
Serous carcinoma	1	0
Malignant mixed Mullerian tumor	2	0
Squamous cell carcinoma	0	13
Neuroendocrine carcinoma	0	1
Adenocarcinoma	0	4
FIGO IA	7	0
FIGO IB	6	1
FIGO IB1	0	3
FIGO IB2	0	2
FIGO IIB	0	5
FIGO IIIB	0	3
FIGO IIIC1	3	0
FIGO IIIC2	2	0
FIGO IVA	0	1
FIGO IVB	1	3

Abbreviations: SEM, standard error of the mean; FIGO, The International Federation of Gynecology and Obstetrics stages

**Fig 3:**
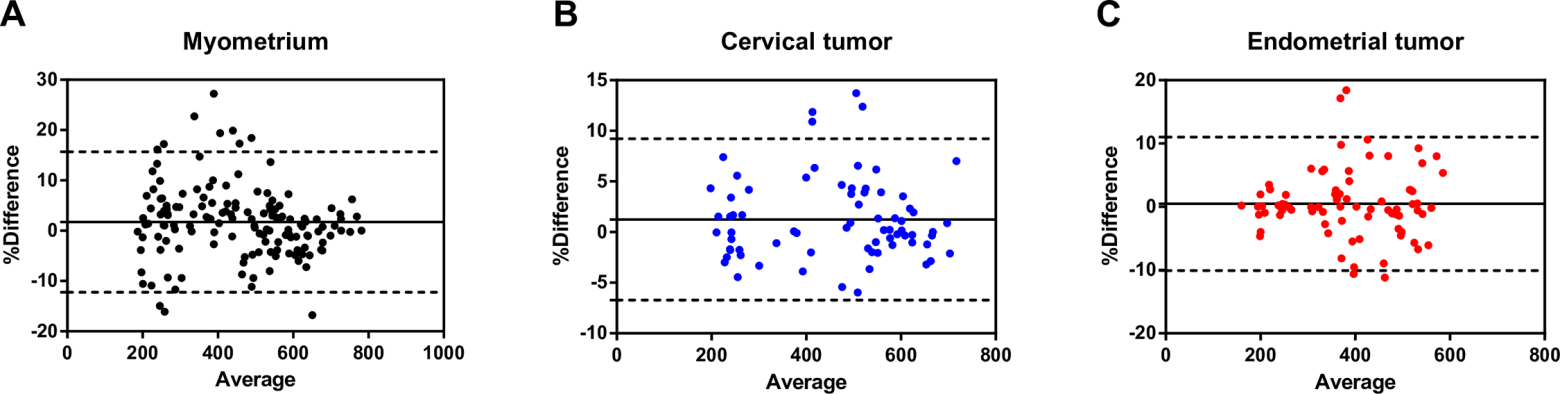
Bland-Altman analysis of agreement in MR signal measurements by two observers regarding the (A) myometrium, (B) tumor tissue from cervical cancer, and (C) tumor tissue from endometrial cancer. The solid center line represents the mean of differences. The top dashed line shows the upper 95% limit of agreement and the bottom dashed line shows the lower 95% limit of agreement.

The selected ROIs for tumor vs. normal myometrium were 59.9 ± 2.0 mm^2^ and 44.5 ± 2.8 mm^2^, respectively. **Fig 3** shows the interobserver variation in the measurement of SI for the normal myometrium, tumor tissue from cervical cancer, and tumor tissue from endometrial cancer. The interobserver agreement rates were 0.957 (0.939–0.955), 0.970 (0.963–0.977) and 0.963 (0.954–0.972) for the measurements of SI in the myometrium, cervical cancer, and endometrial cancer, respectively.

**Fig 4:**
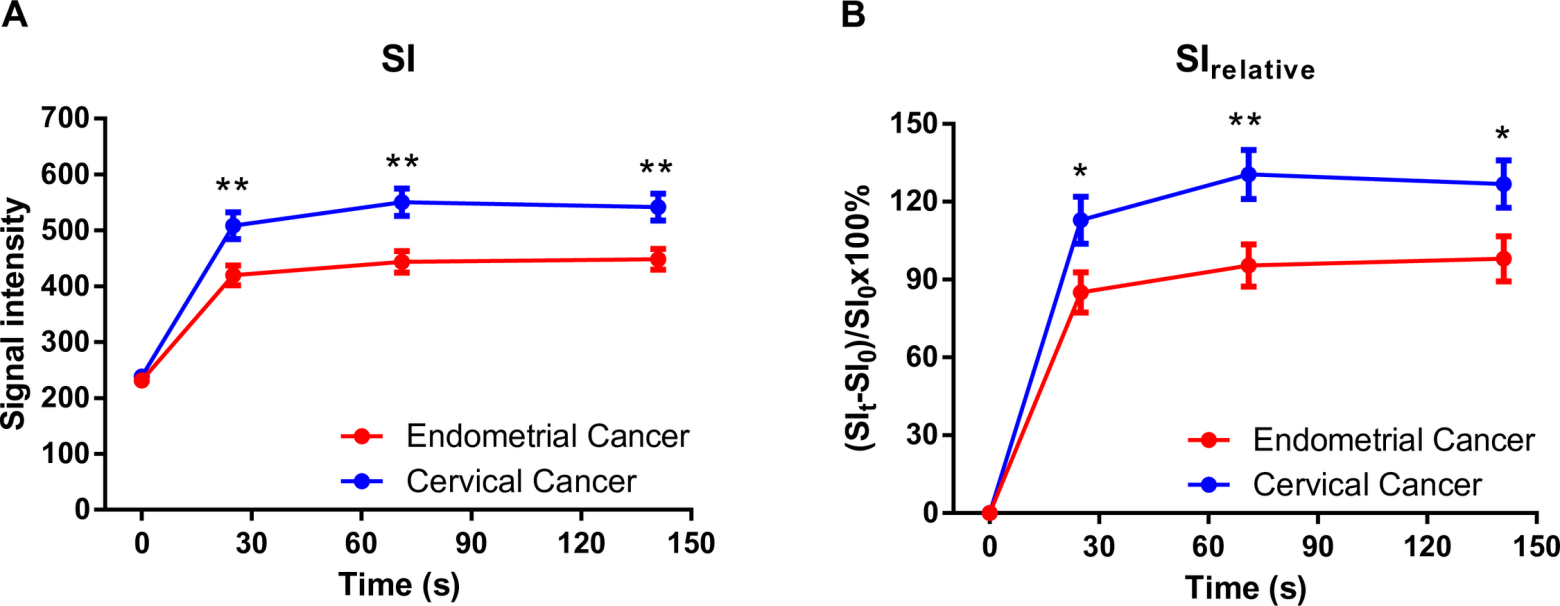
Signal intensity of tumor tissue on MCE-MRI. (A) Signal intensity and (B) relative signal enhancement in the endometrial and cervical cancer prior to (time = 0 s) and after contrast administration. * = p < 0.05, ** = p < 0.01 by unpaired t-test. Data are expressed as mean ± SEM. Abbreviations: SI, signal intensity; SI_0_, pre-contrast SI_t_; SI following contrast administration; SI_relative_, relative signal enhancement.

The measured SI and SI_relative_, for neoplastic tissue of both cervical and endometrial cancer origin, showed rapid initial enhancement and then remained relatively constant **(Fig 4**). However, cervical and endometrial tumor tissues showed significantly different degrees of signal enhancement after contrast administration. The average SI_relative_ for cervical cancer was approximately 30% higher than that of endometrial cancer after contrast administration.

**Fig 5:**
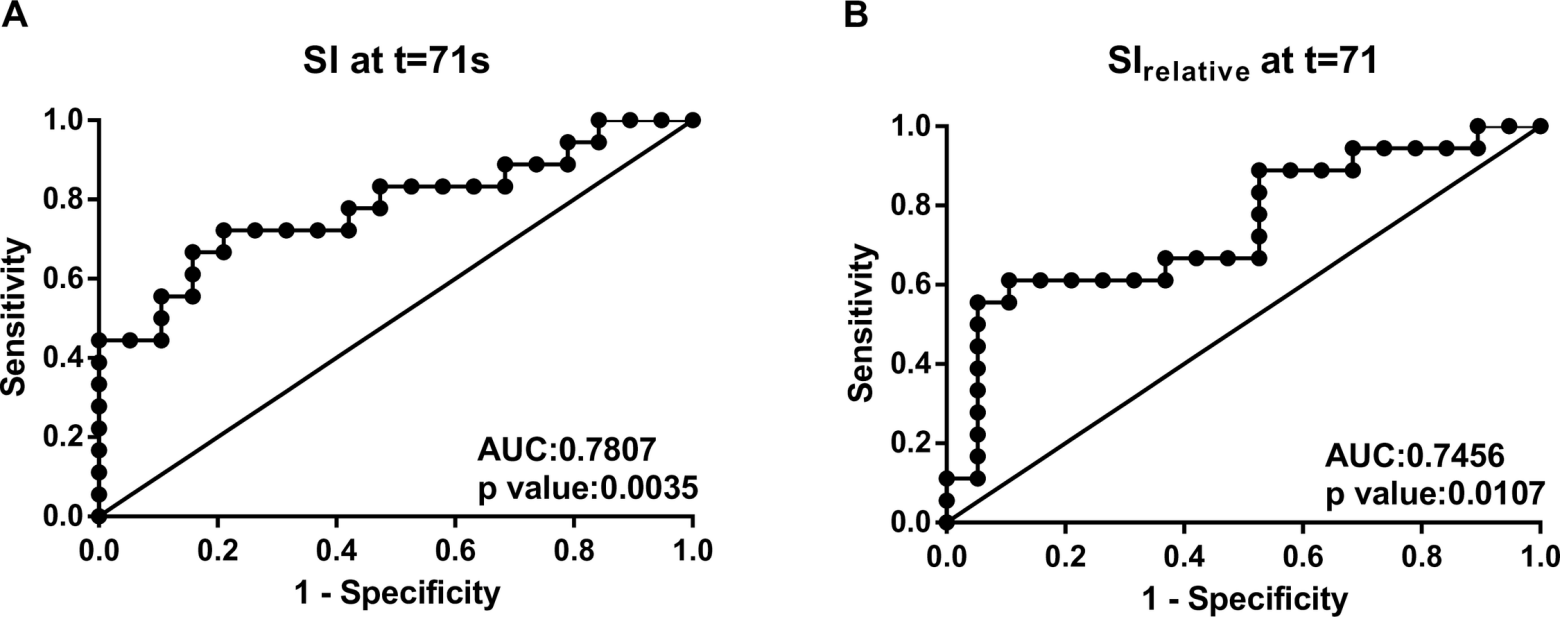
Receiver operating characteristic curves of (A) signal intensity and (B) relative signal enhancement at 71 s after contrast administration for discriminating cervical from endometrial cancer. Abbreviation: AUC, area under the receiver operating characteristic curve.

**Fig 5** showed the ROC analyses which determined the optimal SI and SI_relative_ cutoff values for the separation of cervical from endometrial cancer at 71s after contrast administration. The optimal SI and SI_relative_ cut-off values are 521.4 (72.22% sensitivity, 78.95% specificity) and 122.2% (61.11% sensitivity, 89.47% specificity), respectively (**Fig 5**).

**Fig 6:**
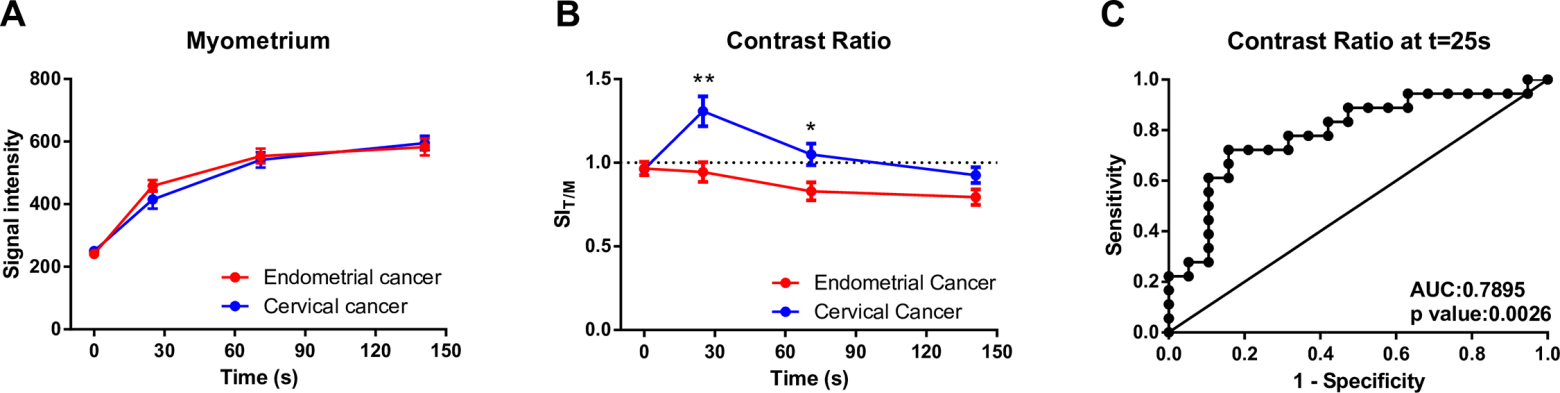
(A) Signal intensity of the normal myometrium in endometrial and cervical cancer on MCE-MRI. (B) Tumor to myometrium contrast ratio in endometrial and cervical cancer on MCE-MRI. (C) Receiver operating characteristic curve of the contrast ratio at 25 s after contrast administration for discriminating cervical from endometrial cancer. Abbreviation: SI_T/M_, SI of tumor divided by SI of the myometrium.

In cases containingboth cervical and endometrial cancer, the non-neoplastic myometrium showed progressive enhancement with a continuous increase in SI throughout the MCE-MRI (**Fig 6A**). There was no difference in the SI of normal myometrium between endometrial and cervical cancer cases prior to and during dynamic contrast enhancement. Generally, tumor tissue from endometrial cancer was relatively hypointense to normal myometrium after contrast administration (**Fig 6B**). In cervical cancer, the SI of tumor tissue was approximately 30% higher than that of normal myometrium at 25 s after contrast enhancement, (**Fig 6B**). Using normal myometrium as an internal reference, SI_T/M_ was the most significant difference between cervical and endometrial tumor at 25 s after contrast enhancement (p = 0.0016, **Fig 6B**), with an optimal sensitivity of 72.22% and a specificity of 84.21% (**Fig 6C**). A slightly higher area under ROC curve (0.7895, **Fig 5B**) was achieved using tumor to myometrium contrast ratio, as compared with that using signal enhancement from baseline (0.7456, **Fig 6C**).

## Discussion

One of the main goals when imaging malignant neoplasms of the uterine corpus and cervix is the accurate assessment of the depth of tumor invasion [13, 14]. Clinically, thin section, high resolution T2W images in the axial oblique and sagittal planes are highly accurate in assessing the depth of myometrial and cervical stromal invasion in gynecologic malignancies. By sampling at more than one time point following contrast administration, MCE-MRI can increase the chance of acquiring optimal tissue contrast between two tissues with different time-intensity curves. MCE-MRI is also used in routine imaging to improve the accuracy of MRI in detecting the extent of tumor invasion into surrounding tissues by endometrial and cervical cancer [7, 8]. The use of the SI value, however, as directly measured from the MR images, is misleading due to the intensity variations in MRI caused by magnetic field inhomogeneity and scanner-related intensity artifacts. Therefore, there is a need to correct for intensity differences in order to perform subsequent imaging analysis. Skeletal muscle is commonly used as an internal reference in MRI. However, there is no suitable skeletal muscle which can act as an internal reference on sagittal MR images of cervical and endometrial cancer.

The MCE-MRI protocol for endometrial and cervical cancer is similar to the triphasic MRI protocols for hepatocellular carcinoma (HCC) surveillance. In the imaging guidelines for HCC diagnosis, the classic enhancement features of HCC are defined when the tumor shows hyperintensity relative to the hepatic parenchyma during the arterial phase and hypointensity relative to the hepatic parenchyma during the venous phase [15]. Similar to the use of non-tumorous liver as an internal reference in the diagnosis of HCC, unaffected myometrium might also be used as a reference for cervical and endometrial cancer. The normal myometrium in this study had a pattern of progressive enhancement with a continuous increase in signal enhancement after gadolinium administration and this result was in agreement with findings from previous studies [16–18].

Although the enhancement pattern of normal myometrium has been well-established, no quantitative study has compared the differences in time-signal intensity curves using the same imaging protocol in endometrial and cervical cancer patients. The uterine myometrium, which consists of smooth muscle cells and interstitial collagen, is not as microscopically homogeneous as is skeletal muscle. In an attempt to provide a rationale for using the normal myometrium as an internal reference in studies involving comparisons of signal enhancement between cervical and endometrial cancer, this study found no significant difference in the enhancement pattern of the normal myometrium in either endometrial or cervical cancer patients.

When compared with healthy myometrium, cervical cancer showed hyperintensity at 25s post contrast injection in the current study. In agreement with our findings, early arterial hypervascularity has been reported to be a favorable feature for cervical cancer in the MRI scoring system proposed by Bourgioti et al. [19]. In their study, hypervascularity on the early arterial phase was diagnosed when tumor enhancement was ≥ normal myometrium by visual comparison. However, lower contrast enhancement of cervical cancer, as compared with normal myometrium, has also been reported by Balleyguier et al. [7]. One possible explanation for the discrepancy in results is that the dynamic scans in these two studies were acquired at different time points. Another possible explanation is that the SI in these two studies was obtained by visual comparison instead of using imaging software measurements. Thus, the simultaneous contrast effect may have affected the degree of brightness, causing contrast illusions [20, 21].

In the current study, the MR signal of the tumor vs. the MR signal of normal myometrium was quantitatively measured by Image J to avoid any visual illusion. Endometrial cancer demonstrated hypointensity relative to normal myometrium post contrast administration in our study. Similar results were observed by other researchers using different dynamic enhanced MRI protocols and visual inspection [8, 11, 22]. Our results support the hypothesis that normal myometrium may act as an internal reference on MRI. With the presence of an internal reference, it may be possible to demonstrate hemodynamic changes in the MCE-MRI dataset of endometrial and cervical cancer using semi-quantitative assessment.

Dynamic contrast-enhanced MRI (DCE-MRI) is an imaging method which acquires consecutive MRI images during and after injection of MR contrast to assess tissue perfusion and tumor angiogenesis [23]. Numerous studies have demonstrated the potential utility of extracted parameters from DCE-MRI for predicting tumor stage, demonstrating lymph node metastasis, and monitoring of treatment response including radiotherapy and anti-angiogenic treatment [24–28]. To acquire further pharmacokinetic parameters from DCE-MRI, a high resolution DCE-MRI protocol should be performed, with a temporal resolution of less than 10 seconds, according to the recommendation of the Quantitative Imaging Biomarkers Alliance committee. Ideally, DCE-MRI data should be acquired with high spatial and temporal resolution to provide both morphological and kinetic information. However, current MRI technologies have limitations and a balance between temporal and spatial resolution is necessary. To maintain acceptable temporal resolution in order to accurately estimate physiological parameters, the currently available DCE-MRI protocols use fast MR sequences to achieve high temporal resolution at the expense of lower image resolution. Furthermore, analysis of DCE-MRI is based on signal enhancement which is altered by the total gadolinium dose, gadolinium injection rate, individual cardiovascular parameters, and the intravenous injection site. To obtain physiological parameters from DCE-MRI, a quantitative DCE-MRI analysis usually involves the use of complex mathematical modeling and post-processing methodology [29–31], which limit its use in daily clinical practice. Therefore, MCE-MRI, rather than DCE-MRI, remains the recommended MRI protocol for HCC and gynecological cancer in clinical practice [7, 8, 19].

In this study, measured SI normalized to baseline and healthy myometrium both represented acceptable performance for discriminating tumor tissues from cervical cancer and endometrial cancer. Our results suggest that both baseline SI and the myometrium are suitable internal references on MCE-MRI, although further investigation with a large sample size is needed to confirm our findings.

This study had several limitations. Normal myometrial tissue may not be visible in large tumors (especially endometrial cancer), limiting the value of the proposed method. As the majority of patients with uterine cancer are diagnosed at an early stage [32], it is very likely that unaffected myometrial tissue is present in most cases. In our study, we did not exclude patients from analysis due to the lack of measurable myometrial tissue. In addition, the ROI was manually defined on the non-necrotic part of the tumor and non-neoplastic part of the outer myometrium. Therefore, the selection of ROI position was challenging and strongly operator-dependent. Also, small and non-measurable lesions were excluded from the study and, therefore, the results of this study may not apply to this subgroup. Finally, subtypes of time-signal intensity curves may have existed within each group of endometrial and cervical cancers. However, the sample size of each subgroup was too small to warrant subgroup analysis.

## Conclusion

Our results suggest MCE-MRI provides added value in the discrimination between cervical cancer and endometrial cancer with an acceptable performance. In addition, this study showed a significant difference in both SI_relative_ and SI_T/M_ between cervical and endometrial cancer after contrast enhancement, yielding similar performance. Therefore, the use of myometrium as an internal reference may provide an alternative method to analyze MCE-MRI of gynecologic cancers.

## Supporting Information

S1 Table Parameters of MR pulse sequences. (DOCX)
