# Supplementary material for: Use of Myometrium as an Internal Reference for Endometrial and Cervical Cancer on Multiphase Contrast-Enhanced MRI
Source: PLoS One. 2016 Jun 21;11(6):e0157820. doi: 10.1371/journal.pone.0157820 (PMC4915709; doi:10.1371/journal.pone.0157820)
Supplement: S1 Table — (DOCX) [file pone.0157820.s001.docx]

# Supporting information captions

S1 Table. Parameters of MR pulse sequences.

| Sequences | Planes | TR  (ms) | TE  (ms) | Flip angles | Thickness (mm) | Gap  (mm) | Averages | FOV (mm) | Matrix | b value |
| --- | --- | --- | --- | --- | --- | --- | --- | --- | --- | --- |
| T2WI | Axial | 4500 | 85 | 150˚ | 5 | 1 | 2 | 240x240 | 256×320 |  |
| T1WI | Axial | 470 | 12 | 150˚ | 5 | 1 | 2 | 240x240 | 256×320 |  |
| T2WI | Sagittal | 3000 | 101 | 90˚ | 3 | 0.6 | 1 | 200x200 | 320×320 |  |
| T2WI | Axial oblique (perpendicular to long axis of uterus) | 4100 | 89 | 150˚ | 3 | 0.4 | 2 | 220x220 | 288$\times320$ |  |
| DWI | Sagittal | 4521 | 84 |  | 3.5 | 0.9 | 4 | 313x334 | 120×128 | 0 /1000 |
| DWI | Axial | 13800 | 83 |  | 3 | 0.4 | 4 | 328x350 | 120×128 | 0 /800 |
| DWI | Axial (diaphragm to pubis) | 7200 | 83 |  | 6 | 1.8 | 3 | 195x240 | 144×192 | 0/50 /1000 |
| T1WI FS | Sagittal | 4.32 | 1.59 | 9˚ | 3 | 0.6 | 1 | 285x380 | 182×320 |  |
| Dynamic | Sagittal | 4.32 | 1.59 | 9˚ | 3 | 0.6 | 1 | 195x240 | 182×320 |  |
| T1WI FS + C | Axial | 6.4 | 1.59 | 9˚ | 3 | 0.6 | 1 | 240x240 | 224×320 |  |
| T1WI FS + C | Axial (diaphragm to iliac crest) | 4 | 1.39 | 9˚ | 4 | 0.8 | 1 | 308x380 | 182×320 |  |
| T1WI FS + C | Axial (iliac crest to pubis) | 3.49 | 1.56 | 9˚ | 5 | 1 | 1 | 285x380 | 216×312 |  |

TR, repetition time; TE, echo time; FOV, field of view; T2WI, T2-weighted image; T1WI, T1-weighted image; DWI, diffusion-weighted image; FS, fat-suppressed; +C, with contrast-enhanced.
